# Supplementary figures and images for: NF-κB is a central regulator of hypoxia-induced gene expression
Source: EMBO Rep. 2025 Nov 26;27(2):416–32. doi: 10.1038/s44319-025-00651-x (PMC12852801; doi:10.1038/s44319-025-00651-x)

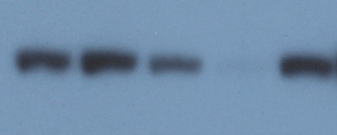

Supplement: Supplementary file 12 — Source data Fig. 6 [file 44319_2025_651_MOESM12_ESM.zip › Figure 6/Figure 6 C/Figure 6 C RelB western blot.tif]

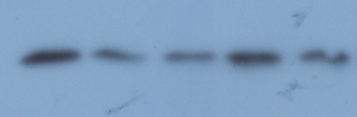

Supplement: Supplementary file 12 — Source data Fig. 6 [file 44319_2025_651_MOESM12_ESM.zip › Figure 6/Figure 6 C/Figure 6 C SOD1 western blot.tif]

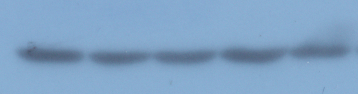

Supplement: Supplementary file 12 — Source data Fig. 6 [file 44319_2025_651_MOESM12_ESM.zip › Figure 6/Figure 6 C/Figure 6 C Actin western blot.tif]

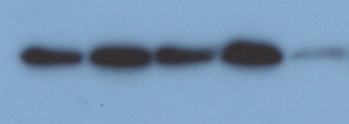

Supplement: Supplementary file 12 — Source data Fig. 6 [file 44319_2025_651_MOESM12_ESM.zip › Figure 6/Figure 6 C/Figure 6 C cRel western blot.tif]

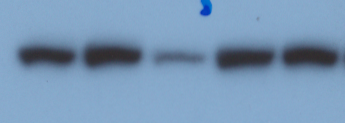

Supplement: Supplementary file 12 — Source data Fig. 6 [file 44319_2025_651_MOESM12_ESM.zip › Figure 6/Figure 6 C/Figure 6 C RelA western blot.tif]

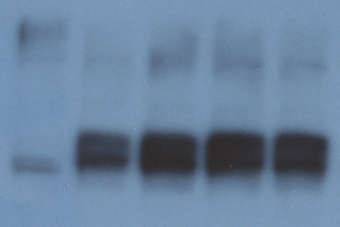

Supplement: Supplementary file 12 — Source data Fig. 6 [file 44319_2025_651_MOESM12_ESM.zip › Figure 6/Figure 6 C/Figure 6 C HIF1A western blot.tif]

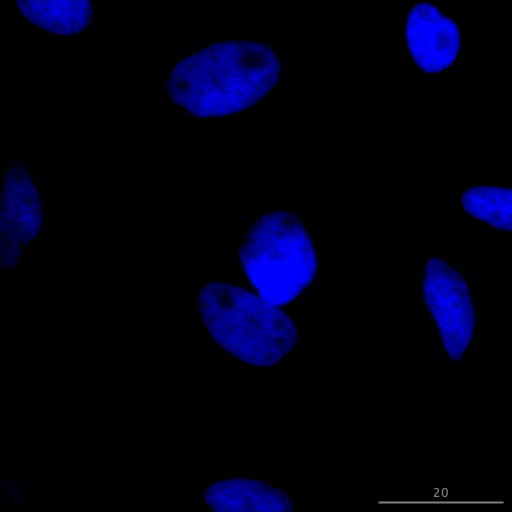

Supplement: Supplementary file 12 — Source data Fig. 6 [file 44319_2025_651_MOESM12_ESM.zip › Figure 6/Figure 6 A/Figure 6 A DAPI_Hpx siRelA.tif]

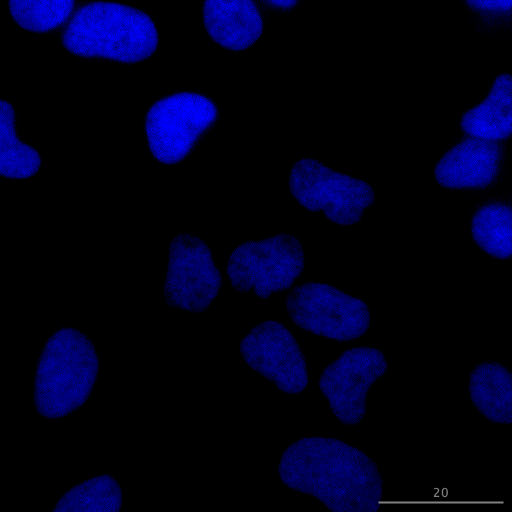

Supplement: Supplementary file 12 — Source data Fig. 6 [file 44319_2025_651_MOESM12_ESM.zip › Figure 6/Figure 6 A/Figure 6 A DAPI_Hpx siRelB.tif]

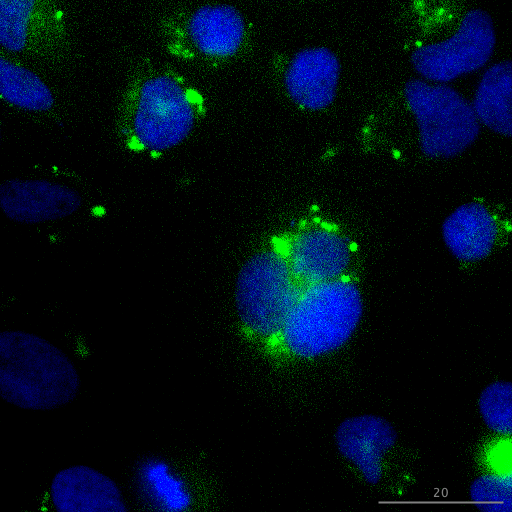

Supplement: Supplementary file 12 — Source data Fig. 6 [file 44319_2025_651_MOESM12_ESM.zip › Figure 6/Figure 6 A/Figure 6 A Merged_Hpx sicRel.tif]

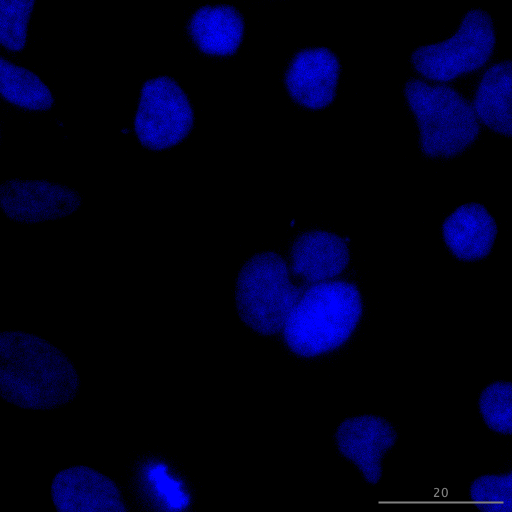

Supplement: Supplementary file 12 — Source data Fig. 6 [file 44319_2025_651_MOESM12_ESM.zip › Figure 6/Figure 6 A/Figure 6 A DAPI_Hpx sicRel.tif]

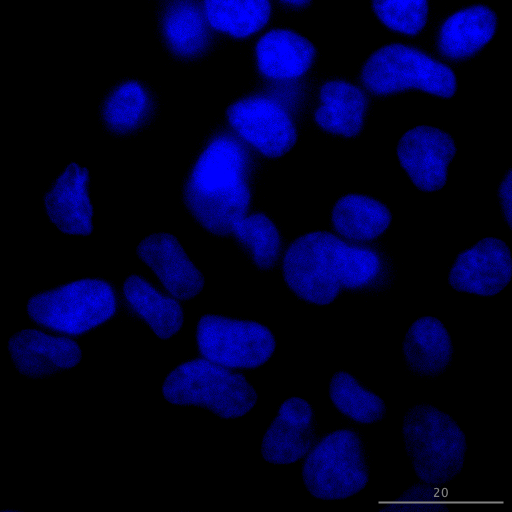

Supplement: Supplementary file 12 — Source data Fig. 6 [file 44319_2025_651_MOESM12_ESM.zip › Figure 6/Figure 6 A/Figure 6 A DAPI_Nx Cntrl.tif]

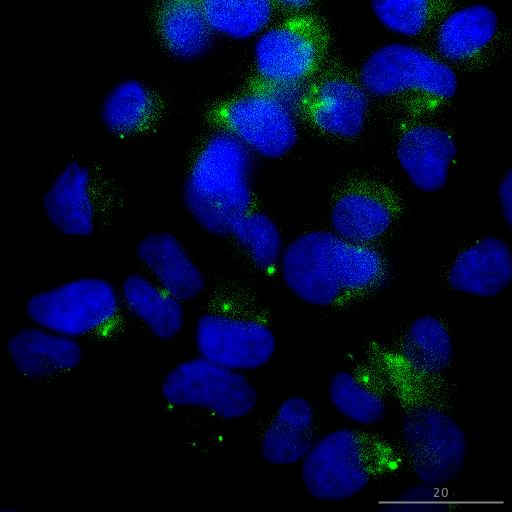

Supplement: Supplementary file 12 — Source data Fig. 6 [file 44319_2025_651_MOESM12_ESM.zip › Figure 6/Figure 6 A/Figure 6 A Merged_Nx Cntrl.tif]

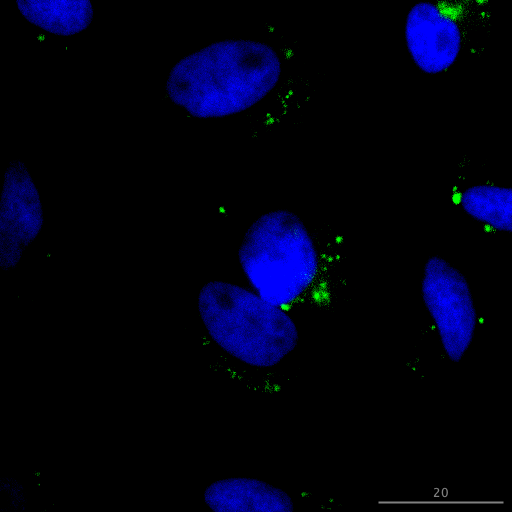

Supplement: Supplementary file 12 — Source data Fig. 6 [file 44319_2025_651_MOESM12_ESM.zip › Figure 6/Figure 6 A/Figure 6 A Merged_Hpx siRelA.tif]

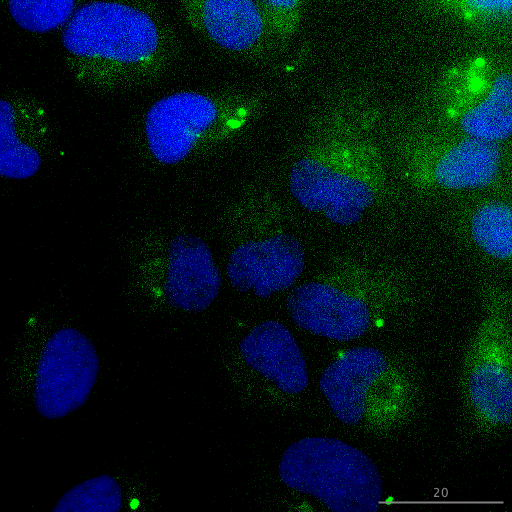

Supplement: Supplementary file 12 — Source data Fig. 6 [file 44319_2025_651_MOESM12_ESM.zip › Figure 6/Figure 6 A/Figure 6 A Merged_Hpx siRelB.tif]

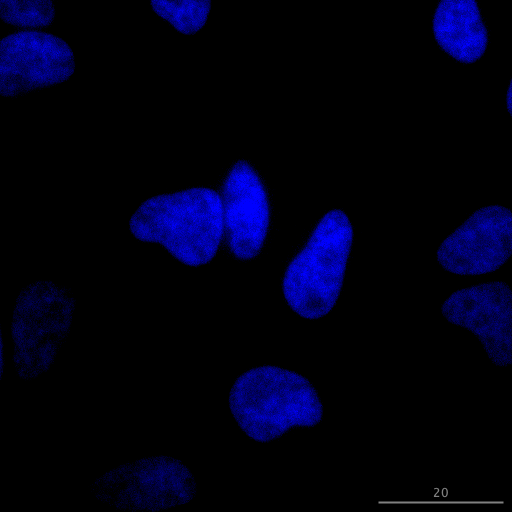

Supplement: Supplementary file 12 — Source data Fig. 6 [file 44319_2025_651_MOESM12_ESM.zip › Figure 6/Figure 6 A/Figure 6 A DAPI_Hpx Cntrl.tif]

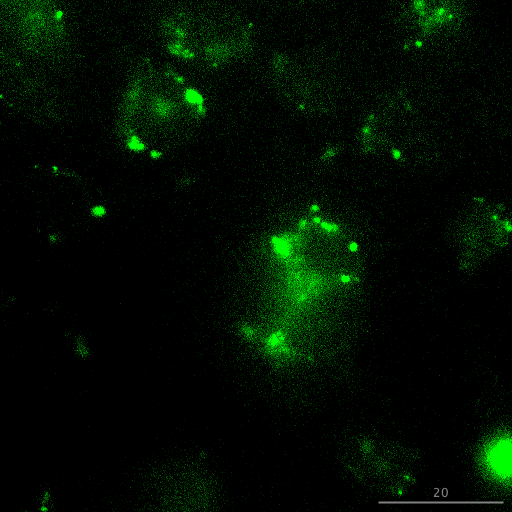

Supplement: Supplementary file 12 — Source data Fig. 6 [file 44319_2025_651_MOESM12_ESM.zip › Figure 6/Figure 6 A/Figure 6 A GFP_Hpx sicRel.tif]

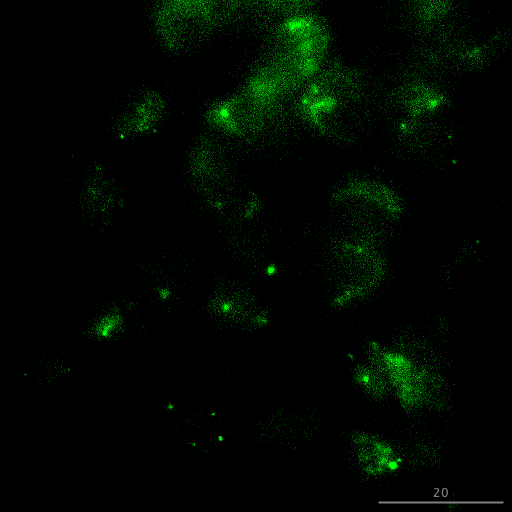

Supplement: Supplementary file 12 — Source data Fig. 6 [file 44319_2025_651_MOESM12_ESM.zip › Figure 6/Figure 6 A/Figure 6 A GFP_Nx Cntrl.tif]

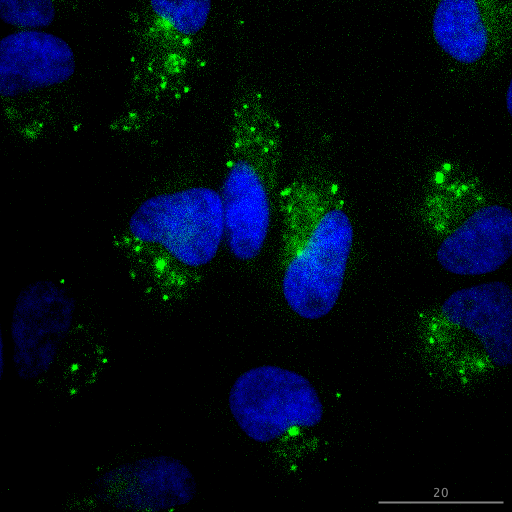

Supplement: Supplementary file 12 — Source data Fig. 6 [file 44319_2025_651_MOESM12_ESM.zip › Figure 6/Figure 6 A/Figure 6 A Merged_Hpx Cntrl.tif]

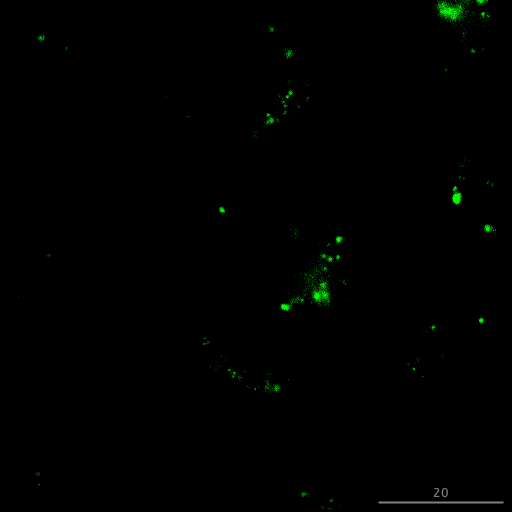

Supplement: Supplementary file 12 — Source data Fig. 6 [file 44319_2025_651_MOESM12_ESM.zip › Figure 6/Figure 6 A/Figure 6 A GFP_Hpx siRelA.tif]

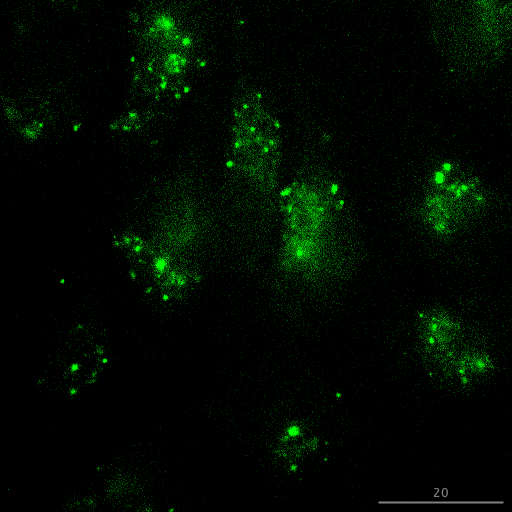

Supplement: Supplementary file 12 — Source data Fig. 6 [file 44319_2025_651_MOESM12_ESM.zip › Figure 6/Figure 6 A/Figure 6 A GFP_Hpx Cntrl.tif]

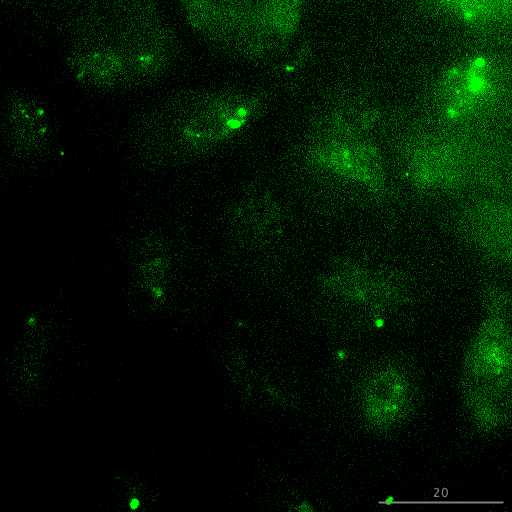

Supplement: Supplementary file 12 — Source data Fig. 6 [file 44319_2025_651_MOESM12_ESM.zip › Figure 6/Figure 6 A/Figure 6 A GFP_Hpx siRelB.tif]

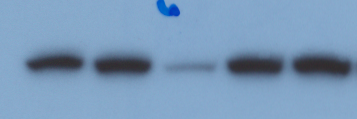

Supplement: Supplementary file 12 — Source data Fig. 6 [file 44319_2025_651_MOESM12_ESM.zip › Figure 6/Figure 6 C/Figure 6 C western blot_biological replicates/Figure 6 C RelA western blot_biological replicate n3.tif]

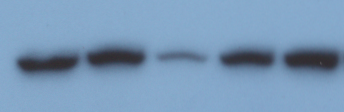

Supplement: Supplementary file 12 — Source data Fig. 6 [file 44319_2025_651_MOESM12_ESM.zip › Figure 6/Figure 6 C/Figure 6 C western blot_biological replicates/Figure 6 C RelA western blot_biological replicate n1.tif]

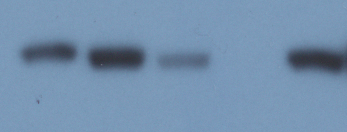

Supplement: Supplementary file 12 — Source data Fig. 6 [file 44319_2025_651_MOESM12_ESM.zip › Figure 6/Figure 6 C/Figure 6 C western blot_biological replicates/Figure 6 C RelB western blot_biological replicate n3.tif]

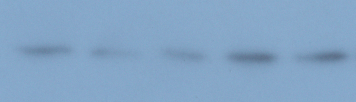

Supplement: Supplementary file 12 — Source data Fig. 6 [file 44319_2025_651_MOESM12_ESM.zip › Figure 6/Figure 6 C/Figure 6 C western blot_biological replicates/Figure 6 C SOD1 western blot_biological replicate n3.tif]

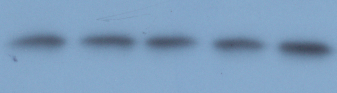

Supplement: Supplementary file 12 — Source data Fig. 6 [file 44319_2025_651_MOESM12_ESM.zip › Figure 6/Figure 6 C/Figure 6 C western blot_biological replicates/Figure 6 C SOD1 western blot_biological replicate n1.tif]

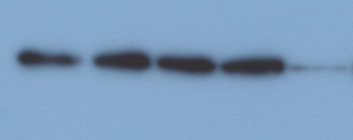

Supplement: Supplementary file 12 — Source data Fig. 6 [file 44319_2025_651_MOESM12_ESM.zip › Figure 6/Figure 6 C/Figure 6 C western blot_biological replicates/Figure 6 C cRel western blot_biological replicate n3.tif]

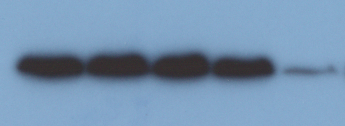

Supplement: Supplementary file 12 — Source data Fig. 6 [file 44319_2025_651_MOESM12_ESM.zip › Figure 6/Figure 6 C/Figure 6 C western blot_biological replicates/Figure 6 C cRel western blot_biological replicate n1.tif]

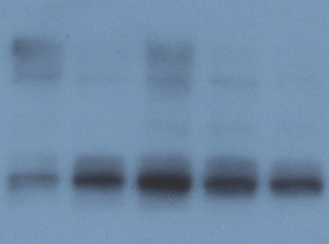

Supplement: Supplementary file 12 — Source data Fig. 6 [file 44319_2025_651_MOESM12_ESM.zip › Figure 6/Figure 6 C/Figure 6 C western blot_biological replicates/Figure 6 C HIF1A western blot_biological replicate n3.tif]

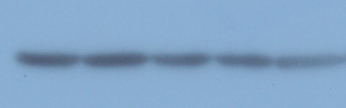

Supplement: Supplementary file 12 — Source data Fig. 6 [file 44319_2025_651_MOESM12_ESM.zip › Figure 6/Figure 6 C/Figure 6 C western blot_biological replicates/Figure 6 C Actin western blot_biological replicate n1.tif]

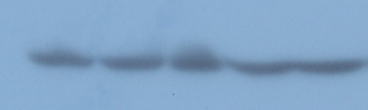

Supplement: Supplementary file 12 — Source data Fig. 6 [file 44319_2025_651_MOESM12_ESM.zip › Figure 6/Figure 6 C/Figure 6 C western blot_biological replicates/Figure 6 C Actin western blot_biological replicate n3.tif]

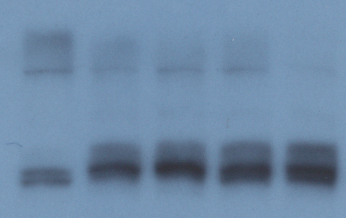

Supplement: Supplementary file 12 — Source data Fig. 6 [file 44319_2025_651_MOESM12_ESM.zip › Figure 6/Figure 6 C/Figure 6 C western blot_biological replicates/Figure 6 C HIF1A western blot_biological replicate n1.tif]

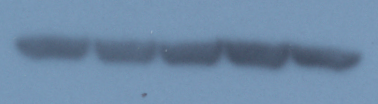

Supplement: Supplementary file 13 — Source data Fig. 7 [file 44319_2025_651_MOESM13_ESM.zip › Figure 7/Figure 7 A/Figure 7 A Actin western blot.tif]

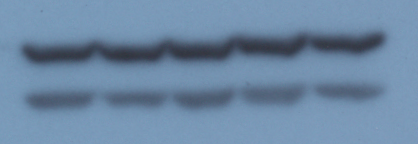

Supplement: Supplementary file 13 — Source data Fig. 7 [file 44319_2025_651_MOESM13_ESM.zip › Figure 7/Figure 7 A/Figure 7 A ATP5A_UQCRC2 western blot.tif]

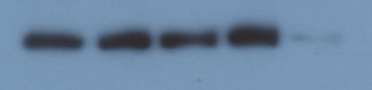

Supplement: Supplementary file 13 — Source data Fig. 7 [file 44319_2025_651_MOESM13_ESM.zip › Figure 7/Figure 7 A/Figure 7 A cRel western blot.tif]

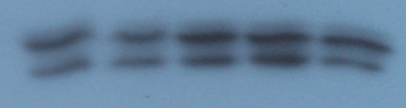

Supplement: Supplementary file 13 — Source data Fig. 7 [file 44319_2025_651_MOESM13_ESM.zip › Figure 7/Figure 7 A/Figure 7 A COX II_NDUFB8 western blot.tif]

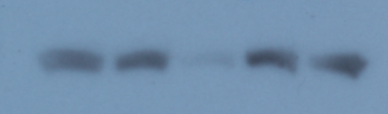

Supplement: Supplementary file 13 — Source data Fig. 7 [file 44319_2025_651_MOESM13_ESM.zip › Figure 7/Figure 7 A/Figure 7 A RelA western blot.tif]

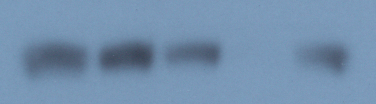

Supplement: Supplementary file 13 — Source data Fig. 7 [file 44319_2025_651_MOESM13_ESM.zip › Figure 7/Figure 7 A/Figure 7 A RelB western blot.tif]

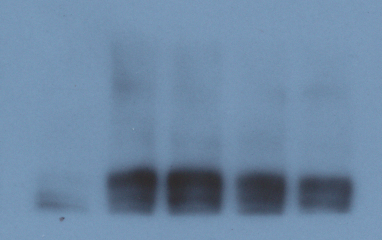

Supplement: Supplementary file 13 — Source data Fig. 7 [file 44319_2025_651_MOESM13_ESM.zip › Figure 7/Figure 7 A/Figure 7 A HIF1A western blot.tif]

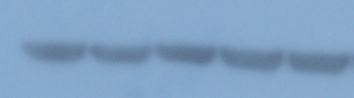

Supplement: Supplementary file 13 — Source data Fig. 7 [file 44319_2025_651_MOESM13_ESM.zip › Figure 7/Figure 7 B/Figure 7 B Actin western blot.tif]

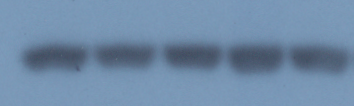

Supplement: Supplementary file 13 — Source data Fig. 7 [file 44319_2025_651_MOESM13_ESM.zip › Figure 7/Figure 7 B/Figure 7 B IDH1 western blot.tif]

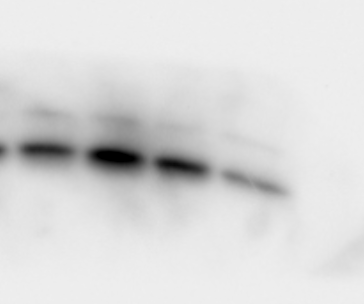

Supplement: Supplementary file 13 — Source data Fig. 7 [file 44319_2025_651_MOESM13_ESM.zip › Figure 7/Figure 7 D/Figure 7 D H3 western blot.tif]

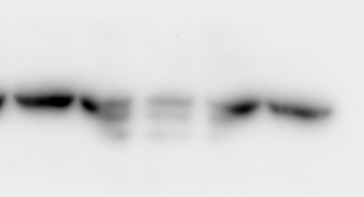

Supplement: Supplementary file 13 — Source data Fig. 7 [file 44319_2025_651_MOESM13_ESM.zip › Figure 7/Figure 7 D/Figure 7 D Actin western blot.tif]

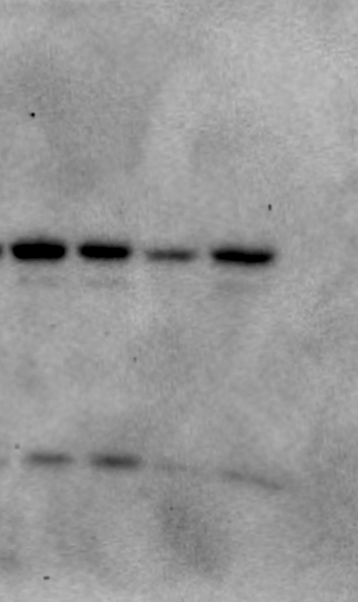

Supplement: Supplementary file 13 — Source data Fig. 7 [file 44319_2025_651_MOESM13_ESM.zip › Figure 7/Figure 7 D/Figure 7 D OxPhos western blot.tif]

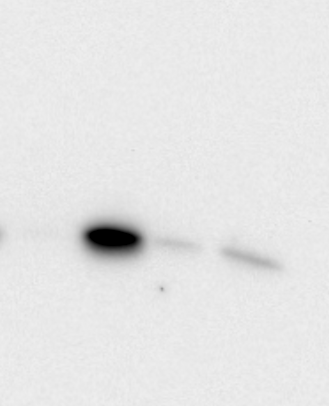

Supplement: Supplementary file 13 — Source data Fig. 7 [file 44319_2025_651_MOESM13_ESM.zip › Figure 7/Figure 7 D/Figure 7 D AcH3 K9_K14 western blot.tif]

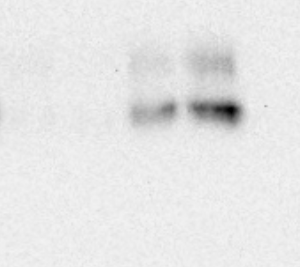

Supplement: Supplementary file 13 — Source data Fig. 7 [file 44319_2025_651_MOESM13_ESM.zip › Figure 7/Figure 7 D/Figure 7 D HIF1A western blot.tif]

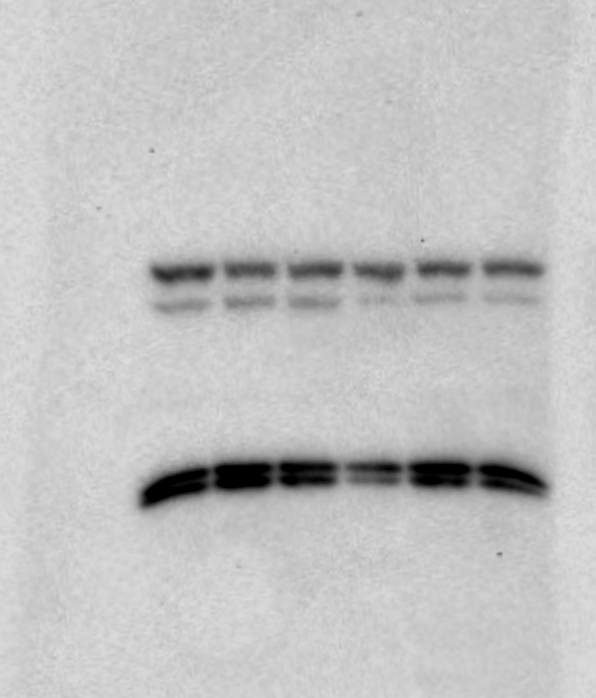

Supplement: Supplementary file 13 — Source data Fig. 7 [file 44319_2025_651_MOESM13_ESM.zip › Figure 7/Figure 7 C/Figure 7 C OxPhos western blot.tif]

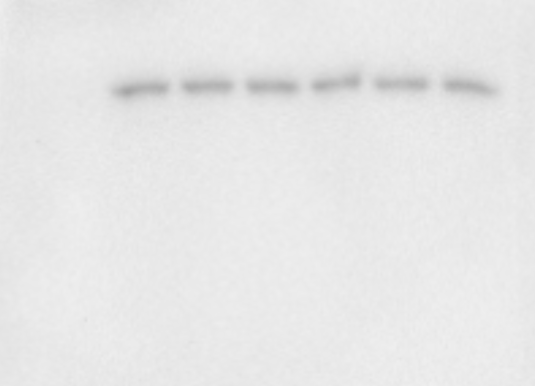

Supplement: Supplementary file 13 — Source data Fig. 7 [file 44319_2025_651_MOESM13_ESM.zip › Figure 7/Figure 7 C/Figure 7 C Actin western blot.tif]

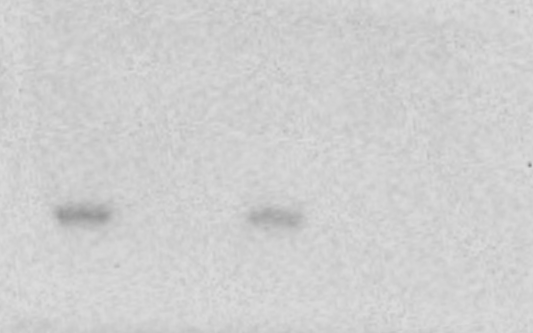

Supplement: Supplementary file 13 — Source data Fig. 7 [file 44319_2025_651_MOESM13_ESM.zip › Figure 7/Figure 7 C/Figure 7 C IKKA western blot.tif]

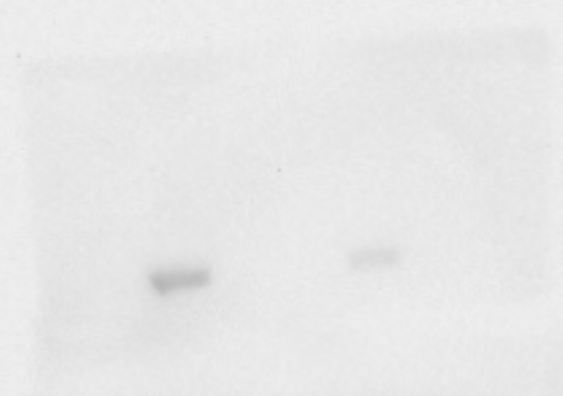

Supplement: Supplementary file 13 — Source data Fig. 7 [file 44319_2025_651_MOESM13_ESM.zip › Figure 7/Figure 7 C/Figure 7 C IKKB western blot.tif]

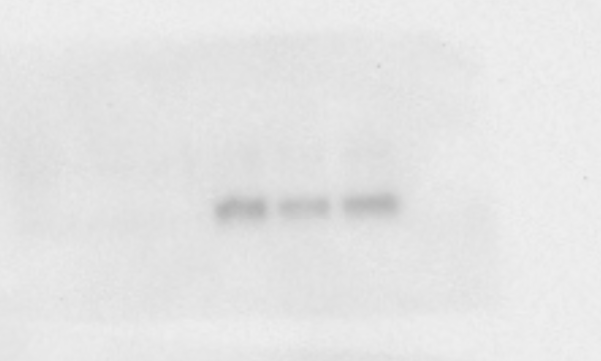

Supplement: Supplementary file 13 — Source data Fig. 7 [file 44319_2025_651_MOESM13_ESM.zip › Figure 7/Figure 7 C/Figure 7 C HIF1A western blot.tif]

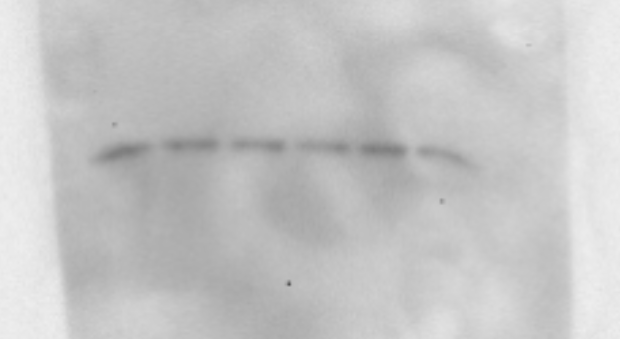

Supplement: Supplementary file 13 — Source data Fig. 7 [file 44319_2025_651_MOESM13_ESM.zip › Figure 7/Figure 7 C/Figure 7 C SOD1 western blot.tif]

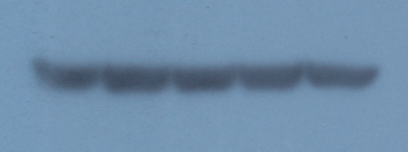

Supplement: Supplementary file 13 — Source data Fig. 7 [file 44319_2025_651_MOESM13_ESM.zip › Figure 7/Figure 7 A/Figure 7 A western blot_biological replicates/Figure 7 A Actin western blot_biological replicate n1.tif]

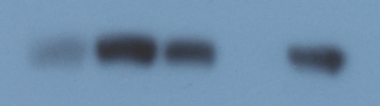

Supplement: Supplementary file 13 — Source data Fig. 7 [file 44319_2025_651_MOESM13_ESM.zip › Figure 7/Figure 7 A/Figure 7 A western blot_biological replicates/Figure 7 A RelB western blot_biological replicate n4.tif]

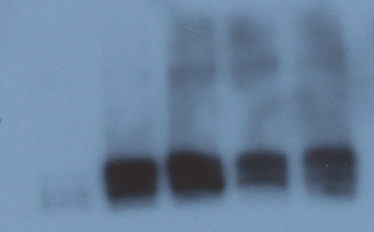

Supplement: Supplementary file 13 — Source data Fig. 7 [file 44319_2025_651_MOESM13_ESM.zip › Figure 7/Figure 7 A/Figure 7 A western blot_biological replicates/Figure 7 A HIF1A western blot_biological replicate n3.tif]

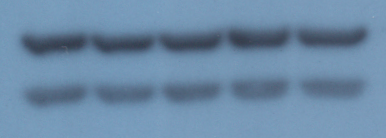

Supplement: Supplementary file 13 — Source data Fig. 7 [file 44319_2025_651_MOESM13_ESM.zip › Figure 7/Figure 7 A/Figure 7 A western blot_biological replicates/Figure 7 A ATP5A_UQCRC2 western blot_biological replicate n3.tif]

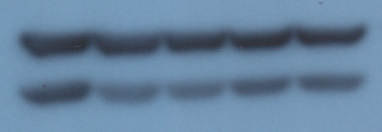

Supplement: Supplementary file 13 — Source data Fig. 7 [file 44319_2025_651_MOESM13_ESM.zip › Figure 7/Figure 7 A/Figure 7 A western blot_biological replicates/Figure 7 A ATP5A_UQCRC2 western blot_biological replicate n1.tif]

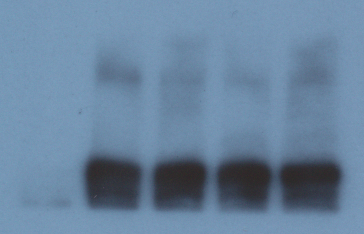

Supplement: Supplementary file 13 — Source data Fig. 7 [file 44319_2025_651_MOESM13_ESM.zip › Figure 7/Figure 7 A/Figure 7 A western blot_biological replicates/Figure 7 A HIF1A western blot_biological replicate n1.tif]

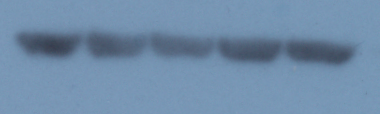

Supplement: Supplementary file 13 — Source data Fig. 7 [file 44319_2025_651_MOESM13_ESM.zip › Figure 7/Figure 7 A/Figure 7 A western blot_biological replicates/Figure 7 A Actin western blot_biological replicate n3.tif]

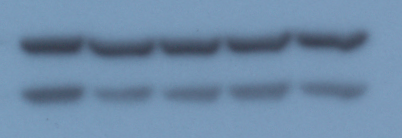

Supplement: Supplementary file 13 — Source data Fig. 7 [file 44319_2025_651_MOESM13_ESM.zip › Figure 7/Figure 7 A/Figure 7 A western blot_biological replicates/Figure 7 A ATP5A_UQCRC2 western blot_biological replicate n4.tif]

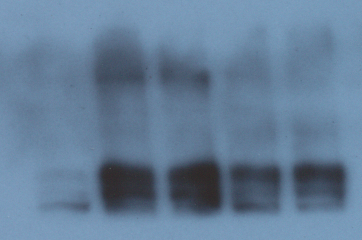

Supplement: Supplementary file 13 — Source data Fig. 7 [file 44319_2025_651_MOESM13_ESM.zip › Figure 7/Figure 7 A/Figure 7 A western blot_biological replicates/Figure 7 A HIF1A western blot_biological replicate n4.tif]

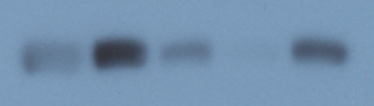

Supplement: Supplementary file 13 — Source data Fig. 7 [file 44319_2025_651_MOESM13_ESM.zip › Figure 7/Figure 7 A/Figure 7 A western blot_biological replicates/Figure 7 A RelB western blot_biological replicate n3.tif]

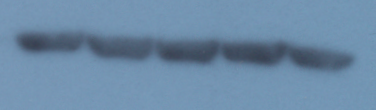

Supplement: Supplementary file 13 — Source data Fig. 7 [file 44319_2025_651_MOESM13_ESM.zip › Figure 7/Figure 7 A/Figure 7 A western blot_biological replicates/Figure 7 A Actin western blot_biological replicate n4.tif]

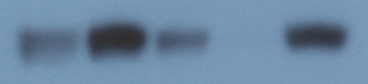

Supplement: Supplementary file 13 — Source data Fig. 7 [file 44319_2025_651_MOESM13_ESM.zip › Figure 7/Figure 7 A/Figure 7 A western blot_biological replicates/Figure 7 A RelB western blot_biological replicate n1.tif]

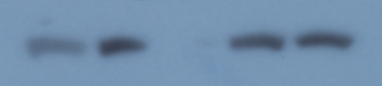

Supplement: Supplementary file 13 — Source data Fig. 7 [file 44319_2025_651_MOESM13_ESM.zip › Figure 7/Figure 7 A/Figure 7 A western blot_biological replicates/Figure 7 A RelA western blot_biological replicate n3.tif]

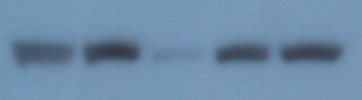

Supplement: Supplementary file 13 — Source data Fig. 7 [file 44319_2025_651_MOESM13_ESM.zip › Figure 7/Figure 7 A/Figure 7 A western blot_biological replicates/Figure 7 A RelA western blot_biological replicate n1.tif]

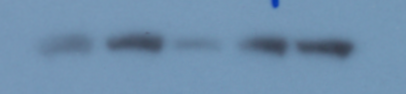

Supplement: Supplementary file 13 — Source data Fig. 7 [file 44319_2025_651_MOESM13_ESM.zip › Figure 7/Figure 7 A/Figure 7 A western blot_biological replicates/Figure 7 A RelA western blot_biological replicate n4.tif]

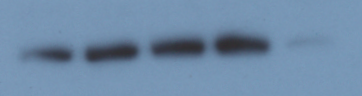

Supplement: Supplementary file 13 — Source data Fig. 7 [file 44319_2025_651_MOESM13_ESM.zip › Figure 7/Figure 7 A/Figure 7 A western blot_biological replicates/Figure 7 A cRel western blot_biological replicate n3.tif]

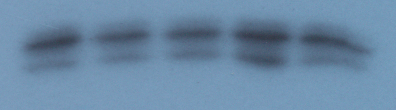

Supplement: Supplementary file 13 — Source data Fig. 7 [file 44319_2025_651_MOESM13_ESM.zip › Figure 7/Figure 7 A/Figure 7 A western blot_biological replicates/Figure 7 A COX II_NDUFB8 western blot_biological replicate n4.tif]

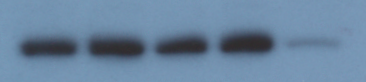

Supplement: Supplementary file 13 — Source data Fig. 7 [file 44319_2025_651_MOESM13_ESM.zip › Figure 7/Figure 7 A/Figure 7 A western blot_biological replicates/Figure 7 A cRel western blot_biological replicate n1.tif]

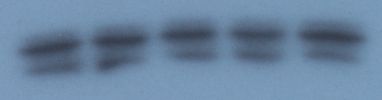

Supplement: Supplementary file 13 — Source data Fig. 7 [file 44319_2025_651_MOESM13_ESM.zip › Figure 7/Figure 7 A/Figure 7 A western blot_biological replicates/Figure 7 A COX II_NDUFB8 western blot_biological replicate n3.tif]

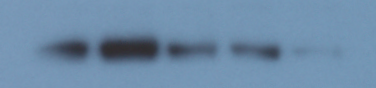

Supplement: Supplementary file 13 — Source data Fig. 7 [file 44319_2025_651_MOESM13_ESM.zip › Figure 7/Figure 7 A/Figure 7 A western blot_biological replicates/Figure 7 A cRel western blot_biological replicate n4.tif]

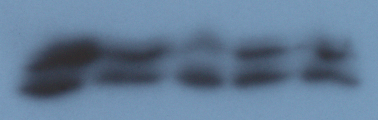

Supplement: Supplementary file 13 — Source data Fig. 7 [file 44319_2025_651_MOESM13_ESM.zip › Figure 7/Figure 7 A/Figure 7 A western blot_biological replicates/Figure 7 A COX II_NDUFB8 western blot_biological replicate n1.tif]

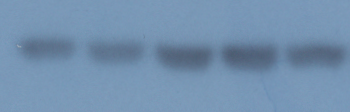

Supplement: Supplementary file 13 — Source data Fig. 7 [file 44319_2025_651_MOESM13_ESM.zip › Figure 7/Figure 7 B/Figure 7 B western blot_biological replicates/Figure 7 B IDH1 western blot_biological replicate n3.tif]

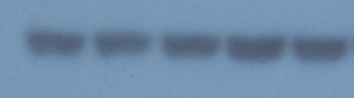

Supplement: Supplementary file 13 — Source data Fig. 7 [file 44319_2025_651_MOESM13_ESM.zip › Figure 7/Figure 7 B/Figure 7 B western blot_biological replicates/Figure 7 B IDH1 western blot_biological replicate n2.tif]

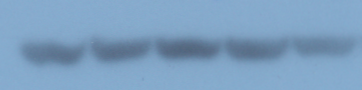

Supplement: Supplementary file 13 — Source data Fig. 7 [file 44319_2025_651_MOESM13_ESM.zip › Figure 7/Figure 7 B/Figure 7 B western blot_biological replicates/Figure 7 B Actin western blot_biological replicate n2.tif]

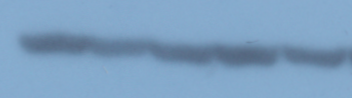

Supplement: Supplementary file 13 — Source data Fig. 7 [file 44319_2025_651_MOESM13_ESM.zip › Figure 7/Figure 7 B/Figure 7 B western blot_biological replicates/Figure 7 B Actin western blot_biological replicate n3.tif]
